# Assessment of the impact of land use in an agricultural catchment area on water quality of lowland rivers

**DOI:** 10.7717/peerj.10564

**Published:** 2021-02-17

**Authors:** Jerzy M. Kupiec, Ryszard Staniszewski, Szymon Jusik

**Affiliations:** Department of Ecology and Environmental Protection, Poznan University of Life Sciences, Poznan, Great Poland, Poland

**Keywords:** Orla River, Agricultural catchment, Nitrate vulnerable zone, Non-point sources of pollution, Macrophytes

## Abstract

In several countries around the world, agricultural land area exceeds 70% (Uruguay 82.6%, Kazakhstan 80.4%, Turkmenistan 72.0%, Great Britain 71.7%, Ukraine 71.6% and others). This poses a serious risk of dissipating nitrates into the aquatic environment in agricultural catchments. The aim of this study was to assess the impact of land use on water quality parameters in an agricultural catchment area. It was decided to select for analysis the catchment of the Orla River (river length of 88 km, catchment area of 1,546 km^2^). The catchment area is predominantly agricultural in character and its entire area has been declared as an agricultural nitrate vulnerable zone (NVZ). A total of 27 survey sites were selected on the main watercourse and its tributaries. Analyses were conducted in the years 2010–2012 to determine physical and chemical parameters of water (pH reaction, conductivity, dissolved oxygen, total nitrogen, organic nitrogen, ammonia nitrogen, nitrates, total and reactive phosphorus) as well as six macrophyte metrics of ecological status assessment (MIR, IBMR, RMNI, MTR, TIM, RI). The average values of most physico-chemical parameters of water quality repeatedly exceeded limits of good ecological status, both in the Orla River and its tributaries. As many as 18 survey sites were classified as moderate ecological status, five sites as poor and only four as good ecological status. The results indicate the impact of land use in the catchment on water conductivity. Differences were observed in the concentrations of biotic components in the main watercourse and its tributaries, and in water quality in the southern part of the catchment in relation to the rest of the study area. This is probably connected with a greater share of forests and surface waters in that area.

## Introduction

We are currently living in the Anthropocene, in which humans have become the primary driving force of changes in the biogeochemical cycle (Crutzen & Stoermer, 2000). This phenomenon is manifested in the degradation of ecosystem structure and alteration of evolutionarily developed processes, such as the water cycle and the cycle of biotic components. At present these phenomena coincide with global demographic, climatic and socio-economic changes, frequently having a detrimental effect on local habitats. Increasing awareness of processes generating global threats promotes integration of knowledge in hydrology and ecology based on the International Hydrological Programme and the UNESCO Man and Biosphere Programme. Effective solution for diffuse pollution from agriculture can be echohydrological approach implemented i.e., in river Pilica basin ( Izydorczyk et al., 2019). It requires close cooperation between scientists (hydrologists, biologists and others), water managers, local authorities and stakeholders working together to achieve good ecological status of waters. Analyses of the condition of the environment, progressing degradation of biodiversity and growing food production costs on the global scale indicate that currently used measures applied to counter these changes are inadequate. In some continents 70% of land consists of a strongly modified agricultural and urbanized landscape. In such a transformed environment in order to ensure sustainable development it is necessary to regulate ecological processes. Water is the primary factor modifying ecosystem evolution. For this reason, in view of the progressing climate crisis and environmental changes the starting point for the regulation of ecological processes is connected with the quantitative approach to the hydrological cycle analyzed for a given catchment.

With population growth and the resulting pressure to satisfy the growing human needs we have been observing an increased intensity of environment use. Human activity not only generates expected products, but also produces waste, including sewage. Not only does sewage pose a serious threat to the natural environment, but the amount of sewage is also directly proportional to the upward trends for the standard of living. Despite new technologies, the Polish countryside suffers from a poorly developed technical infrastructure. This particularly refers to the water and sewage infrastructure and facilities, as well as the waste management system. It results from the scattered distribution of building development in rural areas, lack of environmental awareness on the part of rural populations as well as high investment costs in non-urbanized areas. Appropriate waste and sewage management promotes improvement of sanitary and living conditions for the local communities and the environment (Dolata & Lira, 2009; Gajewska, Kopeć & Obarska-Pempkowiak, 2011). Sewage generated on farms differs in terms of its composition and character. It consists primarily of domestic wastewater, animal waste, leachate from manure storage facilities or green fodder, runoff from fields and farms (Dymaczewski & Sozański, 2002; Goutry, Zajkowski & Wierzbicki, 2009; Kupiec, Oliskiewicz-Krzywicka & Stachowski, 2015). A problem may also be posed by precipitation waters—rainwater and melt waters, as well as infiltration and drainage waters from land, in which reclamation works have been conducted. Sewage may also be generated on farms from washing of farm facilities or paved yards and washing of agricultural machinery. Supplying non-urbanized areas with drinking water and water for farming operations is a basic pre-condition, which needs to be met to ensure development of agricultural and animal production and to improve the standard of living and hygiene for rural communities. Continuous progress of the countryside results in an increased demand and consumption of water along with the resulting increase in the volume of produced sewage. As much as 60% of building development in rural areas of Poland is spatially scattered, with distances between neighboring farmsteads exceeding 45 m. This type of building development is disadvantageous when considering construction of communal water supply and sewerage systems (Wierzbicki & Gromada, 2000; Kaca, 2007). The main problem in rural areas is connected with the diffusion of nutrients (N and P) in the environment. When present in excessive amounts these two nutrients may cause highly adverse changes in natural ecosystems. Farms typically pose a potential threat to the quality of waters in agricultural catchments, which results from high nutrient balances. Unfortunately, it is not the only problem observed. Another highly important, although frequently neglected aspect is connected with a lack of proper manure storage facilities or their poor technical standard (Kupiec & Zbierska, 2007). Nowadays, the needs of best management practice in agriculture are well known across the world (Gharibadousti, Kharel & Stocker, 2019).

## Study area

**Figure 1:**
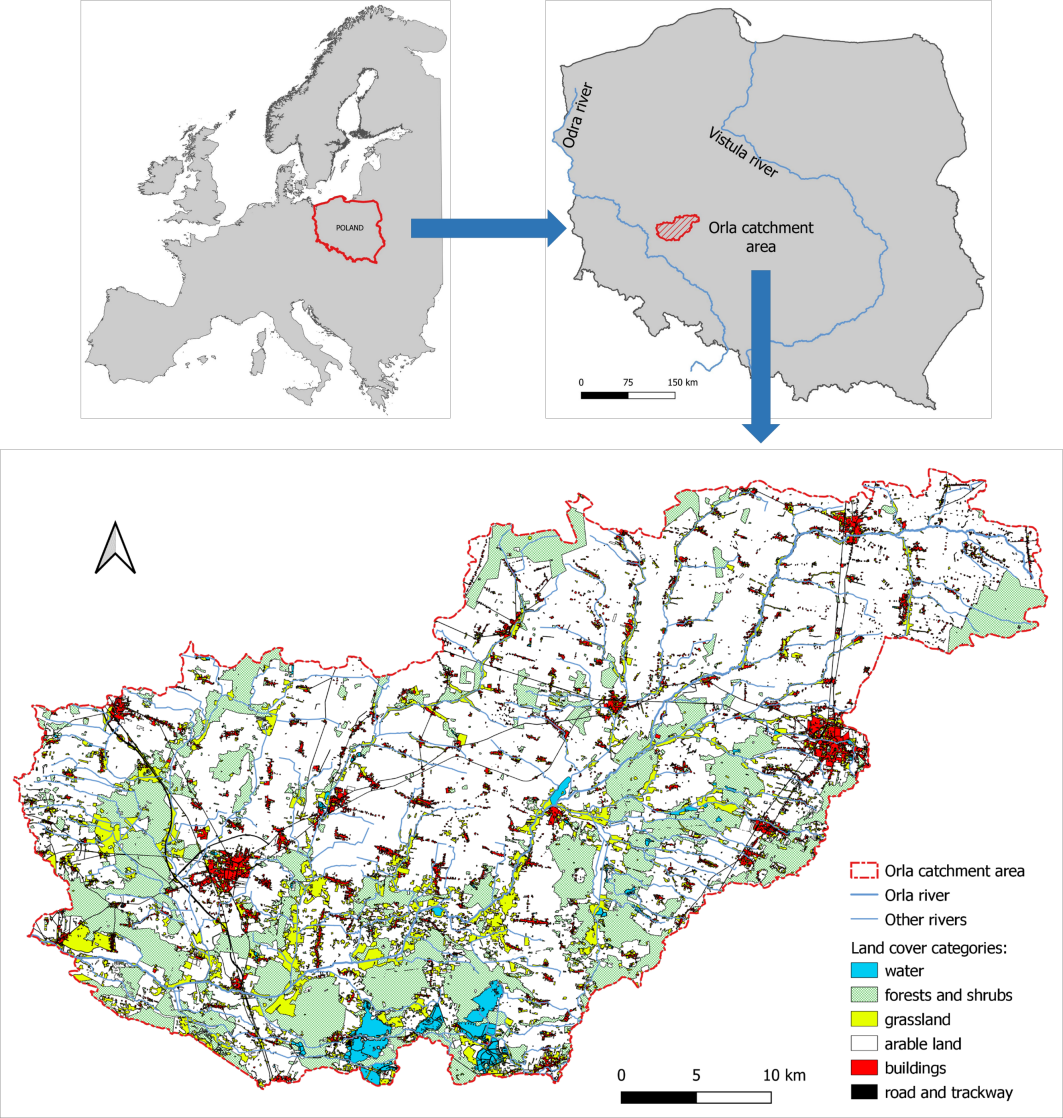
Location and land use structure in the Orla catchment.

**Figure 2:**
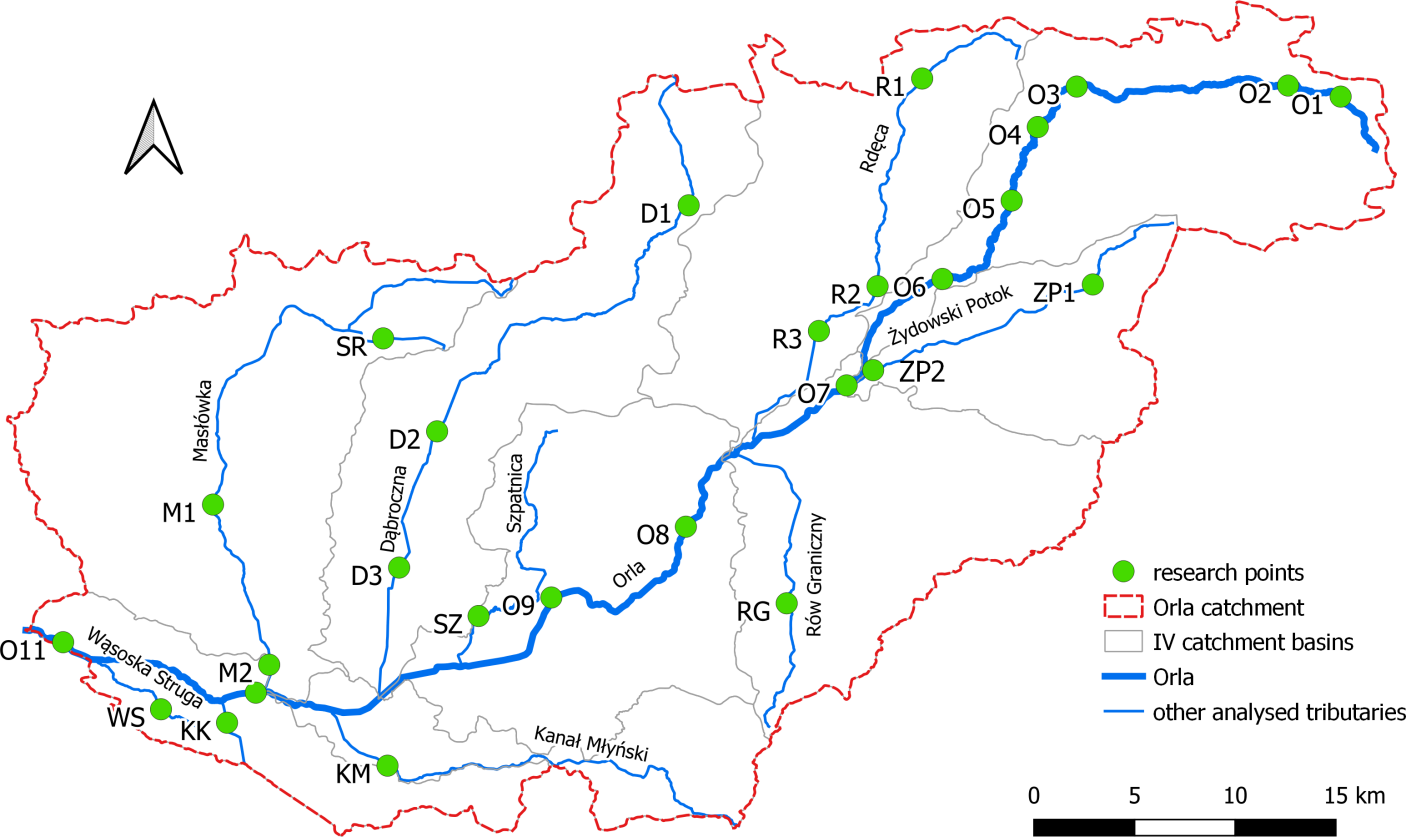
Location of testing sites in the Orla river catchment (site codes as in Table 2). O1 - Orla (Koźminiec), O2 - Orla (Wyki), O3 - Orla (Staniew), O4 - Orla (Skałów),O5 - Orla (Unisław), O6 - Orla (Kuklinów), O7 - Orla (Baszków), O8 - Orla (Dubin), O9 - Orla (Sowy), O10 - Orla (Chodlewo), O11 - Orla (Wąsosz), R1 - Radęca (Bułaków), R2 - Radęca (Wyganów), R3 - Radęca (Kobylin), SZ - Szpatnica (Dębionka), D1 - Dąbrocznia (Gębice), D2 - Dąbrocznia (Miejska Górka), D3 - Dąbrocznia (Stwolno), SR - Szurkowski (Rów Szurkowo), M1 - Masłówka (Żylice), M2 - Masłówka (Korzeńsko), ZP1 - Żydowski Potok (Bożacin), ZP2 - Żydowski Potok (Baszków), RG - Rów Graniczny (Gogołowice), KM - Kanał Młyński ( Baranowice), KK- Kanał Książęcy (Chodlewo), WS - Wąsoska Struga (Unisławice).

The aim of this study was to assess the impact of land use on water quality parameters in an agricultural catchment area. Investigations were conducted in the catchment of the Orla River in western Poland (Fig. 1). The names and codes of water bodies within the Orla catchment are presented in Table 1. In terms of the administrative division the analyzed river is located at the boundary of two provinces: the Wielkopolskie and the Dolnośląskie, 7 counties: the Krotoszyn, Gostyń, Leszno, Rawicz, Pleszew, Milicz and Góra counties, and 22 communes. A total of 27 survey sites were selected on the main watercourse and its tributaries (Fig. 2).

The Orla is a right bank tributary of the Barycz, spanning 88 km in length and with the catchment area of 1,546 km^2^. It flows in the South Wielkopolska Lowland and in the Milicz-Głogów Depression. It flows from the Kalisz Upland near the village of Budy, then transects the Żmigród Basin and flows into the Barycz in the town of Wąsosz. Major towns in the catchment include Koźmin Wielkopolski, Jutrosin, Korzeńsko and Wąsosz. The main tributaries of the Orla are:

• left bank: the Czarna Woda, Żydowski Potok, Borownica, Orla Leniwa and the Wilczyna
• right bank: the Radęca, Szpatnica, Dąbrocznia and the Masłówka.

There is lack of lakes in the catchment and there are few man-made reservoirs, e.g., in Miejska Górka (Malta Lake), Rozstępniewo (Zbiornik “Rozstępniewo”), Pakosław (Zbiornik “Pakosław”), Jutrosin (Zbiornik “Jutrosin”) and a complex of ponds “Stawy Milickie”. They are located mainly in the southern part of the catchment. The lake density index for the analyzed area is 1.2% (the national average for Poland is 0.9%).

**Table 1:** The studied catchment area covers 9 surface water bodies.

Name of the surface water body	European code of surface water body
Dąbrocznia	PLRW600010146699
Kanał Bachorzec	PLRW6000101467265
Kanał Książęcy	PLRW600010146923
Kanał Młyński	PLRW6000111467299
Masłówka	PLRW60001014689
Orla to Radęca	PLRW60001014639
Orla to Radęca to Barycz	PLRW60001114699
Wąsoska Struga	PLRW60001014696
Wilczyna	PLRW60001014658

The Orla catchment is typically agricultural in character (Fig. 1), as arable land accounts for as much as 64.3% total area, while grassland amounts to 8.8%. Forests cover as little as 21.5% catchment area (at the national average for Poland of 29.8%). Small forest complexes consist mainly of pine coniferous forests. Differences in land use are observed between catchment parts located in individual provinces. The share of arable land in the Wielkopolskie province is 77%, while that of forested area is 16%. The catchment area located in the Dolnośląskie province is characterized by a much lesser share of arable land (58%), while that of forests is much greater (30%). The analyzed catchment has many protected areas, which are found primarily in the southern part of the catchment.

Over the last 70–80 years slight changes have been observed in the land use of the catchment. Analyses of archival maps from 1934 (Messtischblatt in a 1:25000 scale) in comparison with the present state showed a decrease in the share of arable land from 70.6% (in 1934) to 64.3% (in 2016). Arable land predominates in the central and north-eastern parts of the catchment, which did not change over the investigated period. Forested areas in 1934 accounted for 16.4% catchment area, while in 2016 their share increased to 21.5%. Forest complexes are located mainly in the southern and western parts of the catchment. At present they are to a considerable extent covered by various spatial nature conservation forms. Analyses of the permanent grassland area in the investigated years showed that its share decreased from 9.4% to 8.8%. Permanent grassland areas are typically located along watercourses and extend across large forested areas. The catchment may also be classified as poorly urbanized, with Rawicz and Krotoszyn being two most important towns. The other towns are scattered throughout the catchment area. The total share of developed land in the catchment is 4.1% and in relation to 1934 it increased by 0.7%. In 1934 the area of surface waters was as little as 3.58 km^2^ (0.2%) and over the several decades it increased to 21.7 km^2^ (1.4%).

The Orla has been classified sensitive to nitrate pollution from agricultural sources (NSA - Nitrate Sensitive Area), while the catchment has been designated as vulnerable to nitrates from agricultural sources (NVZ - Nitrate Vulnerable Zone) (Council of Ministers of Poland, 2017; Governor of the Dolnośląskie Voivodship, 2017). This results from the European Council Directive 91/676/EEC of 12 December, 1991 and its implementation in Poland.

**Figure 3:**
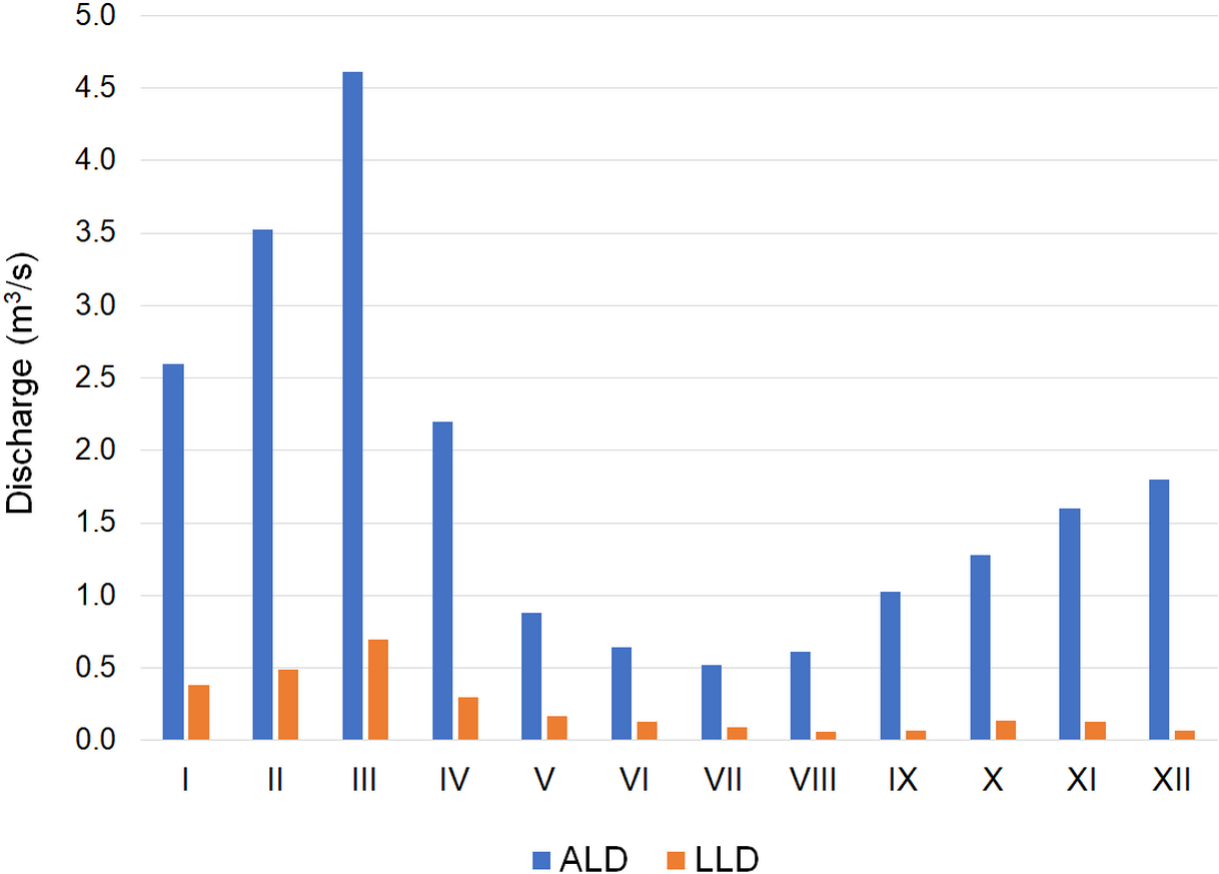
Monthly variation in flows from the multi-annual period of 1981–2010 on the Orla River in the Korzeńsko cross-section.

A river gauge on the Orla is located in Korzeńsko, which roughly corresponds to site O10 (Orla—Chodlewo). Flows from the multi-annual period of 1981–2010 are as follows: averaged of average observed discharges AAD = 4.457 m^3^/s, average discharge from the lowest ALD = 0.315 m^3^/s and lowest observed discharge LOD = 0.060 m^3^/s (Hobot et al., 2012). At the same time considerable monthly variability of flows was observed, with the greatest flows recorded in March: average discharge from the lowest observed ALD = 4.613 m^3^/s, the lowest from lowest discharges LLD = 0.700 m^3^/s, while they were lowest in July: ALD = 0.517 m^3^/s, LLD = 0.060 m^3^/s (Fig. 3).

Within the Orla catchment there are 12 municipal wastewater and 39 industrial sewage discharge points. Overall approx. 2.394 million m^3^/year sewage are discharged to river waters in this catchment, which accounts for approx. 1.7% AAD, 24.1% ALD at the Korzeńsko river gauge. The total pollutant loads amount to BOD_5_—22.582 t O_2_/year, COD—129.797 t O_2_/year, suspended matter—24.612 t/year and chlorides—0.532 t/year.

It was decided to select for analyses the sites located in agriculturally utilized areas in order to exclude the direct effect of other pollution sources on the research results. Investigations were conducted in the years 2010-2012, comparable in terms of prevalent weather conditions and stable in terms of other factors which may potentially modify the physical and chemical, biological or hydromorphological status of the area.

## Methods

In this work, spatial analyzes were performed using the following data sources:

• Database of Topographic Objects (BDOT10k) - national database of spatial data with the detail corresponding to the topographic map in the scale 1: 10,000, the official source of information on land use. The database was created in the years 2012–2013 on the basis of technical guidelines contained in the Regulation of the Minister of Internal Affairs and Administration of November 17, 2011 (Journal of Laws of 2011, No. 279, item 1,642). BDOT10k was used to assess the land use structure of the sub-catchments included in the Orla catchment area and to prepare a map (data valid for 2016).
• Map of the Hydrographic Division of Poland (MPHP10k) - a spatial database with the detail corresponding to the topographic map in the scale 1: 10,000, that is the basis for the official division on the hydrographic of Poland. The database was created on the basis of technical guidelines contained in the Regulation of the Council of Ministers of December 28, 2017 (Journal of Laws of 2017, item 2505). MPHP10k was used to delimit the boundaries of the sub-catchment areas included in the Orla catchment area and to prepare the map.
• Database ”Intakes and Discharges”, being part of the geodatabase made under the project ”Identification of pressures in water regions and river basin districts”, commissioned by the National Water Management Authority. The above-mentioned source of data systematically gathers information covering data of water permits for water intake and discharge of waters and sewage to surface waters. Based on this data the total volume of sewage was calculated together with the total pollutant loads discharged to watercourses located within the Orla catchment.

### Physico-chemical analyses

The pH reaction, conductivity and oxygen concentration were measured at all the sites during field work. Total phosphorus, soluble reactive phosphates (SRP) and nitrates were analysed in the laboratory using the standard Hach-Lange methodology (HachCompany, 2013).

• pH reaction - determined electrometrically on site,
• conductivity—determined electrometrically on site,
• dissolved oxygen - recorded using the sensor method on site,
• total nitrogen—according to Kjeldahl (N org + N-NH_4_ ) + N-NO_2_ + N-NO_3_,
• ammonia nitrogen - by colorimetry with the Nessler reagent,
• nitrates - by the cadmium reduction method,
• total phosphorus - evaluated using persulfate digestion and acid molybdate spectrophotometry,
• soluble reactive phosphorus (filtered - using acid molybdate spectrophotometry).

### Macrophyte surveys

The study of plants in rivers was carried out using the Macrophyte Method for River Assessment, which is the official Polish method of macrophyte monitoring (Szoszkiewicz et al., 2020), in line with the recommendations of the European Committee for Standardization (European Committee for Standardization, 2002). Aquatic plants were surveyed along river stretches 100 m in length. The survey included a list of species and estimated ground cover of plants. Based on the macrophyte database, six macrophyte metrics were calculated, namely the Polish MIR - Macrophyte Index for Rivers (Szoszkiewicz et al., 2020), French IBMR - Indice Biologique Macrophytique en Rivière (Haury et al., 2006), British MTR - Mean Trophic Rank (Holmes et al., 1999) and RMNI - River Macrophyte Nutrient Index (Willby, Pitt & Phillips, 2009), German RI - Reference Index (Schaumburg et al., 2004) and TIM - Trophäe-Index Macrophyten (Schneider, Krumpholz & Melzer, 2000). These indices reflect river degradation, especially eutrophication level (concentration of total phosphorous).

### Statistical analysis

The analysis was preceded by estimating normal distribution of data using the Shapiro–Wilk W test. This test showed that the distribution of data is significantly right-skewed and therefore data transformation was carried out (ln *x* + 1). In the next step, statistical analyses were utilized to find relationships between environmental variables and macrophyte indices using Spearman’s rank correlation. The nonparametric test was selected due to the significant right-skewed distribution of data (even after data transformation).

Canonical ordination analysis for linking the composition of macrophytes to environmental variables was carried out using CANOCO for Windows (Ter Braak & Šmilauer, 2002). Preliminary Detrended Correspondence Analysis (DCA) on the biological data revealed that the gradient length was 3.011 of standard deviations. In the next step direct Canonical Correspondence Analysis (CCA) with forward variable selection was used. Rare taxa were excluded from the analysis (less than four times occurrence). The statistical signi?cance of the relationships between taxa and environmental variables were evaluated using the Monte Carlo permutation test (499 permutations).

## Results

**Figure 4:**
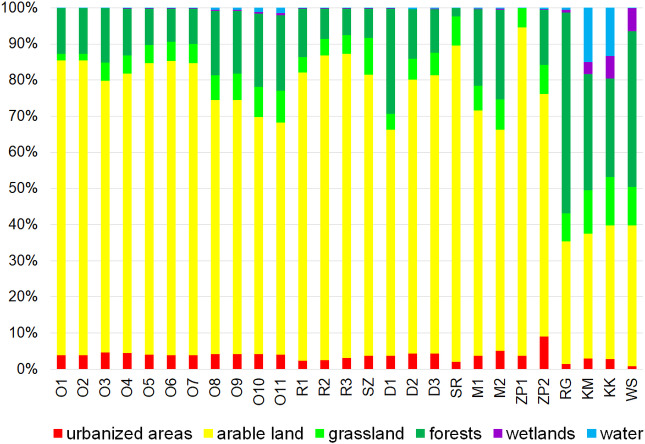
Land use structure in the catchment testing sites (site codes as in Table 2).

The catchment use structure in cross-sections at the outflow of most testing sites shows a considerable dominance of arable land (typically >70%) as well as a very small share of forests and shelterbelts (generally <15%). The share of urbanized areas in catchments usually does not exceed 5% (Fig. 4). In terms of land use four small watercourses are distinguished: Rów Graniczny (RG), Kanał Młyński (KM), Kanał Książęcy (KK) and Wąsoska Struga (WS), located in the southern part of the Orla catchment, mostly in the Dolina Baryczy Landscape Park. Their catchments are characterized by a much greater share of forests and shelterbelts, on average reaching 50%, as well as a considerable share of wetlands and surface waters.

The condition of waters in the Orla catchment is not satisfactory. Underground waters tested in 2015 at the NVZ monitoring site in Szkaradowo showed mean annual concentrations at 103.2 mg NO_3_/dm^3^ (Regional Environmental Protection Inspectorate, REPI, 2015). In 2017 this concentration increased to 121.4 mg NO_3_/dm^3^, while in 2018 it was 113.9 mg NO_3_/dm^3^ (Regional Environmental Protection Inspectorate, REPI, 2017; Regional Environmental Protection Inspectorate, REPI, 2018).

Water quality tests conducted by REPI in the years 2006–2015 in the Orla catchment showed that waters of the Orla and its tributaries are strongly eutrophied. Eutrophication is caused by excessive concentrations of nitrates, total nitrogen and total phosphorus. The primary effect on water quality in the catchment is exerted by diffuse pollutants. Concentrations of nitrogen compounds during a year are subjected to considerable fluctuations, from low to very high values. This is connected with the frequent low water stages in the watercourses (Regional Environmental Protection Inspectorate, REPI, 2007; Melcer & Olejnik, 2006;  Regional Environmental Protection Inspectorate, REPI, 2016).

This research also confirmed the strong eutrophication of the waters of the Orla River and its tributaries (Table 3). The average values of most physico-chemical parameters of water frequently exceeded the limits of good ecological status, both in the Orla River and its tributaries. Only the average values of nitrate nitrogen were in good ecological condition. The largest exceeding concerned orthophosphates (on average 10.7 times in the Orla River and 10.4 times in its tributaries) and ammonia nitrogen (exceedances of 6 times and 12 times). Table 3 also shows the acceptable concentrations of nutrients in water, corresponding to the limit values of good ecological status (Journal of Laws 2019, item 2149) and the values corresponding to the reference conditions for Polish small sandy lowland rivers (Jusik et al., 2015). Physical and chemical parameters investigated in this study differed between the tributaries and the main watercourse. In the case of physical parameters the differences were slight, but in the case of chemical parameters differences between mean and maximum values were marked. In the tributaries maximum values were much greater for most parameters. In turn, mean values for total phosphorus and nitrates were higher in the main watercourse. In the case of total nitrogen higher mean values were recorded for the tributaries (Table 3).

**Table 2:** The list of testing sites.

River code	River name	Site name	Latitude (N)	Longitude (E)
O1	Orla	Koźminiec	51°49′18.02″	17°36′02.28″
O2	Orla	Wyki	51°49′34.17″	17°33′45.33″
O3	Orla	Staniew	51°49′25.46″	17°24′37.17″
O4	Orla	Skałów	51°48′19.05″	17°22′58.75″
O5	Orla	Unisław	51°46′20.03″	17°21′55.04″
O6	Orla	Kuklinów	51°44′11.55″	17°19′00.41″
O7	Orla	Baszków	51°41′38.46″	17°15′40.90″
O8	Orla	Dubin	51°37′23.46″	17°08′12.59″
O9	Orla	Sowy	51°35′24.06″	17°02′30.13″
O10	Orla	Chodlewo	51°32′37.77″	16°49′54.98″
O11	Orla	Wąsosz	51°33′49.33″	16°41′34.36″
R1	Radęca	Bułaków	51°49′26.02″	17°18′19.07″
R2	Radęca	Wyganów	51°43′57.41″	17°16′12.92″
R3	Radęca	Kobylin	51°42′43.21″	17°13′43.84″
SZ	Szpatnica	Dębionka	51°34′51.15″	16°59′23.71″
D1	Dąbrocznia	Gębice	51°45′14.91″	16°05′24.50″
D2	Dąbrocznia	Miejska Górka	51°36′31.20″	16°56′20.93″
D3	Dąbrocznia	Stwolno	51°36′05.41″	16°55′55.24″
SR	Szurkowski Rów	Szurkowo	51°42′12.83″	16°54′57.19″
M1	Masłówka	Żylice	51°37′37.83″	16°47′50.18″
M2	Masłówka	Korzeńsko	51°33′51.69″	16°50′22.87″
ZP1	Żydowski Potok	Bożacin	51°44′08.08″	17°25′30.01″
ZP2	Żydowski Potok	Baszków	51°41′33.70″	17°15′49.71″
RG	Rów Graniczny	Gogołowice	51°35′24.49″	17°12′37.91″
KM	Kanał Młyński	Baranowice	51°30′46.63″	16°55′39.77″
KK	Kanał Książęcy	Chodlewo	51°31′38.32″	17°48′46.99″
WS	Wąsoska Struga	Unisławice	51°31′55.57″	16°46′40.71″

**Table 3:** Physical and chemical parameters of waters in testing sites.

Parameter	Physical and chemical parameters of water							
	pH	conductivity	P_react_PO${}_{4}^{3-}$	P_total_P	N-NO_3_	N-NH_4_	N_total_	O_2_
		mScm^−1^	mgdm^−3^					
Main watercourse								
Mean	7.76	0.96	2.96	1.27	1.80	2.41	8.65	3.19
Minimum	7.42	0.72	0.21	0.32	0.10	0.29	3.55	0.82
Maximum	8.86	1.26	7.65	2.60	6.00	10.89	18.02	5.97
Exceeding the limit value for class II	–	1.4×	10.7×	3.9×	0.9×	6.0×	2.6×	2.4×
Tributaries								
Mean	7.27	0.80	2.89	0.86	0.69	4.77	10.79	2.83
Minimum	7.13	0.39	0.15	0.04	0.00	0.30	2.33	0.00
Maximum	8.40	1.37	9.35	3.89	4.30	15.67	32.20	6.87
Exceeding the limit value for class II	–	1.2x	10.4x	2.6x	0.4x	12.0x	3.3x	2.7x
Ecological status class limits for polish small sandy lowland rivers (Journal of Laws 2019, item 2149)								
I class	–	0.42	0.18	0.52	1.10	0.14	2.00	8.9
II class	–	0.69	0.28	1.01	2.00	0.40	3.30	7.6
Reference condition for polish small sandy lowland rivers (Jusik et al., 2015)								
Mean	7.84	0.46	0.23	0.43	0.25	0.08	–	–
Standard deviation	0.32	0.18	0.14	0.24	0.31	0.08	–	–

During the analyses as many as 90 macrophyte taxa with 9 structural algae, 1 moss, 2 pteridophytes, 39 dicotyledons and 39 monocotyledons were identified. Due to the typical lowland character of the surveyed rivers (laminar current, sandy and silty bottom substrate) vascular plants were the dominant common taxa with a considerable share of *Phalaris arundinacea* (85% of sites), *Sparganium emersum* (67%), *Lemna minor* (67%), *Callitriche sp.* (52%), *Alisma plantago-aquatica* (48%), *Solanum dulcamara* (48%) and *Rorippa amphibia* (45%). All the above-mentioned species are perceived as very common in eutrophic waters of Polish Lowlands (i.e., *Potamogeton pectinatus*). An interesting, rare species in rivers is *Cladium mariscus*, found during the study in two sites (SR—Szurkowski Rów and KM—Kanał Młyński). Among algae the most frequent were *Cladophora sp.*, *Rhizoclonium sp.* and *Vaucheria sp.* (found respectively in 30% of the surveyed sites). The only moss found was *Leptodictyum riparium* (at site R3—Radęca Kobylin).

Based on the results obtained during field analyses, which supplement information on the macrophyte species composition and their cover, six macrophyte indices of the ecological status were calculated (MIR, IBMR, RMNI, MTR, TIM, RI). The calculated macrophyte indices confirm the strong eutrophication of the studied river sections and the problem of failure to achieve good ecological status (Haury et al., 2006; Holmes et al., 1999; Schaumburg et al., 2004; Schneider, Krumpholz & Melzer, 2000; Szoszkiewicz et al., 2020; Willby, Pitt & Phillips, 2009) (Table 4). For example values of MIR (Szoszkiewicz et al., 2020) ranged from 22.4 at the Masłówka Korzeńsko site (M2) to 49.3 at the Rów Graniczny site (RG). A total of 18 sites were classified as moderate ecological status, 5 as poor and 4 as good (Journal of Laws 2019, item 2149). The worst ecological status was observed in the Dąbrocznia River, Masłówka and Żydowski Potok (poor\moderate status), which indicates their strong eutrophication. The number of taxa in the investigated sites ranged from 5 (O3—Orla Staniew) to 25 (O2—Orla Wyki), at the mean of 15 (Table 4).

**Table 4:** Macrophyte indices in testing sites.

Value	No of species	Macrophyte cover (%)	MIR	IBMR	RMNI	MTR	TIM	RI
Main watercourse (Orla river)								
Median	15	29	31.7	8.63	7.80	32.7	2.94	−9.9
Minimum	5	8	28.5	7.30	6.70	27.9	2.00	−29.4
Maximum	25	81	41.1	10.42	8.14	38.0	3.06	27.6
Tributaries								
Median	14	32	28.5	8.85	7.66	32.2	2.83	4.6
Minimum	8	7	22.4	4.79	6.58	23.0	2.60	−51.9
Maximum	21	190	49.3	11.36	8.12	41.8	3.11	44.4

The degree of river channel overgrowth by aquatic vegetation was analysed based on the total macrophyte cover. This parameter varied greatly; however, the river channels were typically overgrown at over 1/3 cross-section. The macrophyte cover ranged from 7.2% (R2—Radęca Wyganów) up to 190.2% (KK—Kanał Książęcy), at the mean of 42.7% (Table 4). In three cases the cover was >100%, which resulted from a very strong development of macrophytes in several layers simultaneously, including strong development of pleustophytes, mainly *Lemna minor*, on the water surface). Typically sites located in locations shaded by trees and shrubs growing on river banks and blocking sunlight are characterized by poor vegetation growth and limited total macrophyte cover, whereas segments of rivers in open areas, characterized by full insolation of the river channel, are strongly overgrown by vegetation.

**Figure 5:**
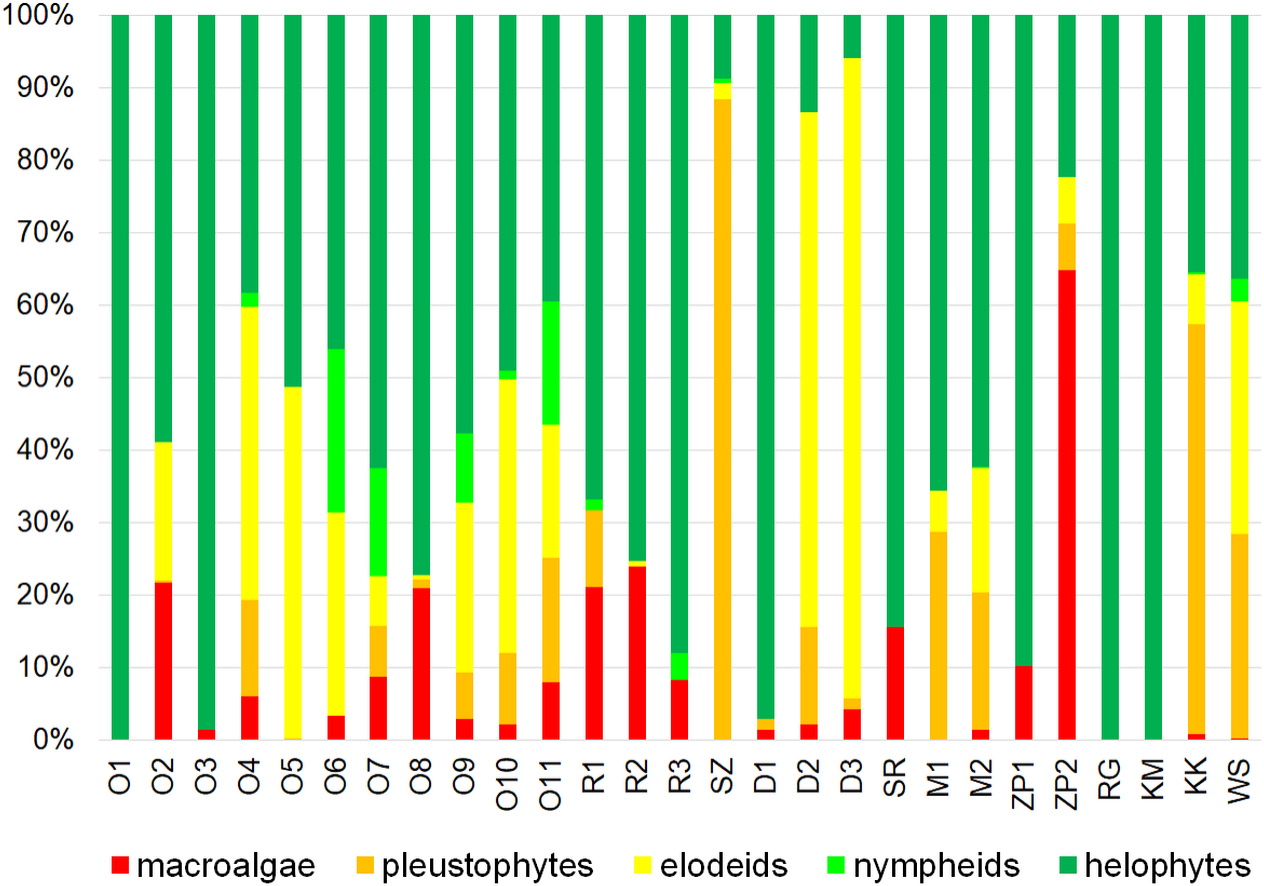
The share of ecological groups in macrophyte cover in investigated sites (site codes as given in Table 2).

In terms of the share of ecological groups in the total macrophyte cover helophytes (emergent plants) predominated in most sites. They accounted for a mean 60% total macrophyte cover (Fig. 5). Helophytes were the only or almost exclusively present (>95% macrophyte cover) in five sites: Orla Koźminiec (O1), Orla Staniew (O3), Dąbrocznia Gębice (D1), Rów Graniczny (RG) and Kanał Młyński (KM). The Dąbrocznia at the Miejska Górka (D2) and Żylice (D3) sites was a river dominated by elodeids (stem plants that complete their entire lifecycle submerged, or with only their flowers above the waterline). A considerable share in the vegetation cover was found for such species as *Potamogeton pectinatus* and *Ceratophyllum submersum*. In three sites a considerable share in the vegetation cover was recorded for pleustophytes (*Lemna minor*), accounting for 30 up to almost 90% share among all macrophytes, while in one macroscopic algae were dominant (65% share).

**Figure 6:**
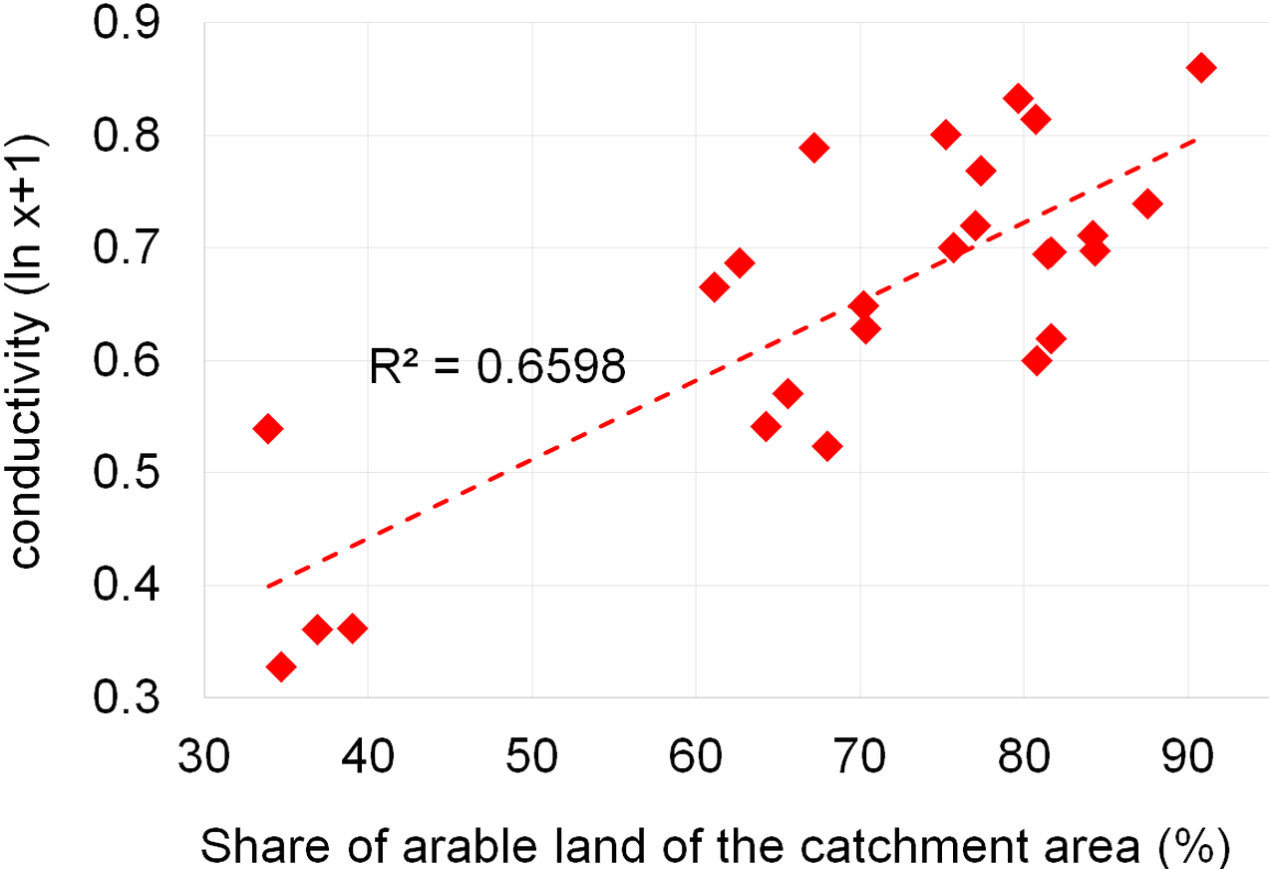
Dependencies between the arable land and electrolytic conductivity of water.

**Figure 7:**
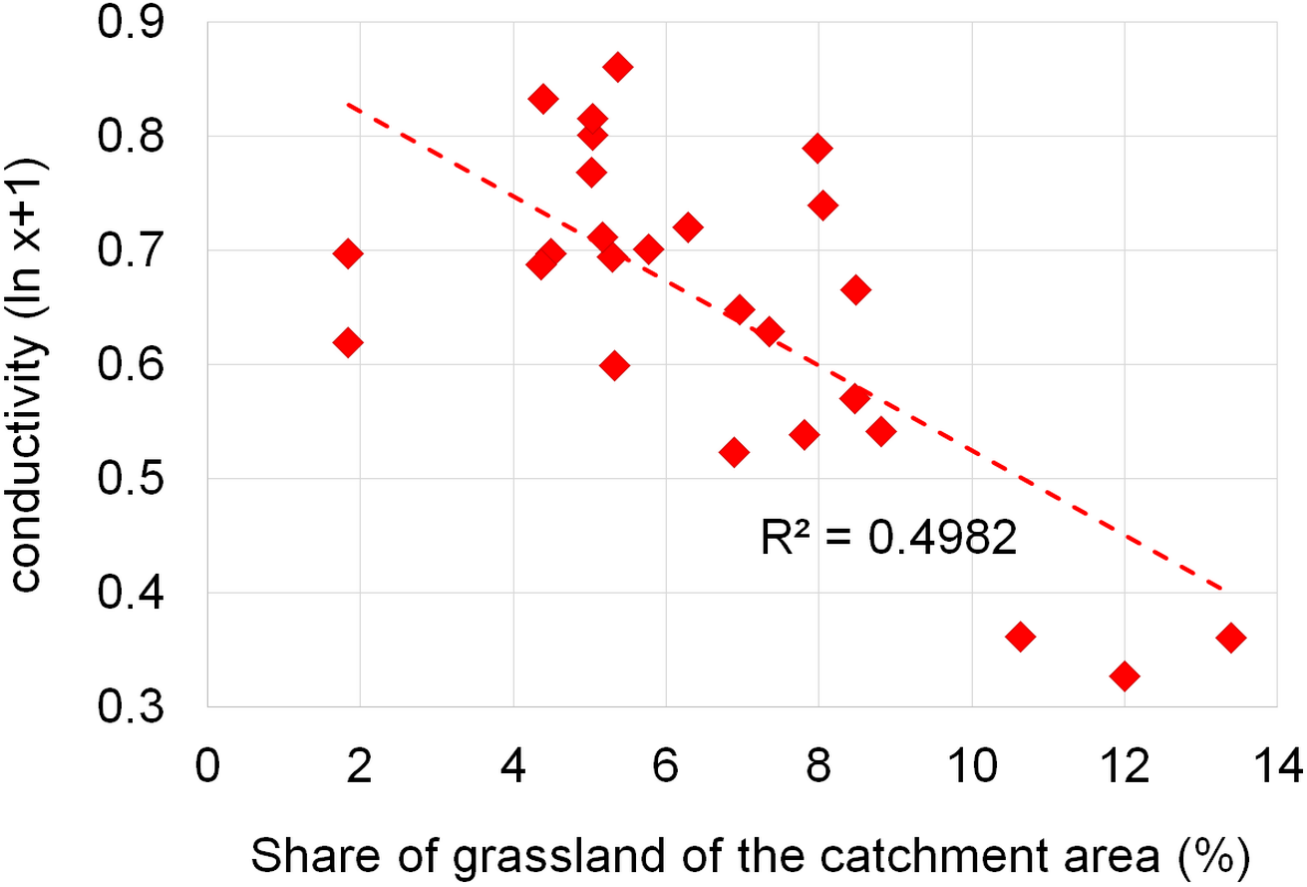
Dependencies between the grassland and electrolytic conductivity of water.

**Figure 8:**
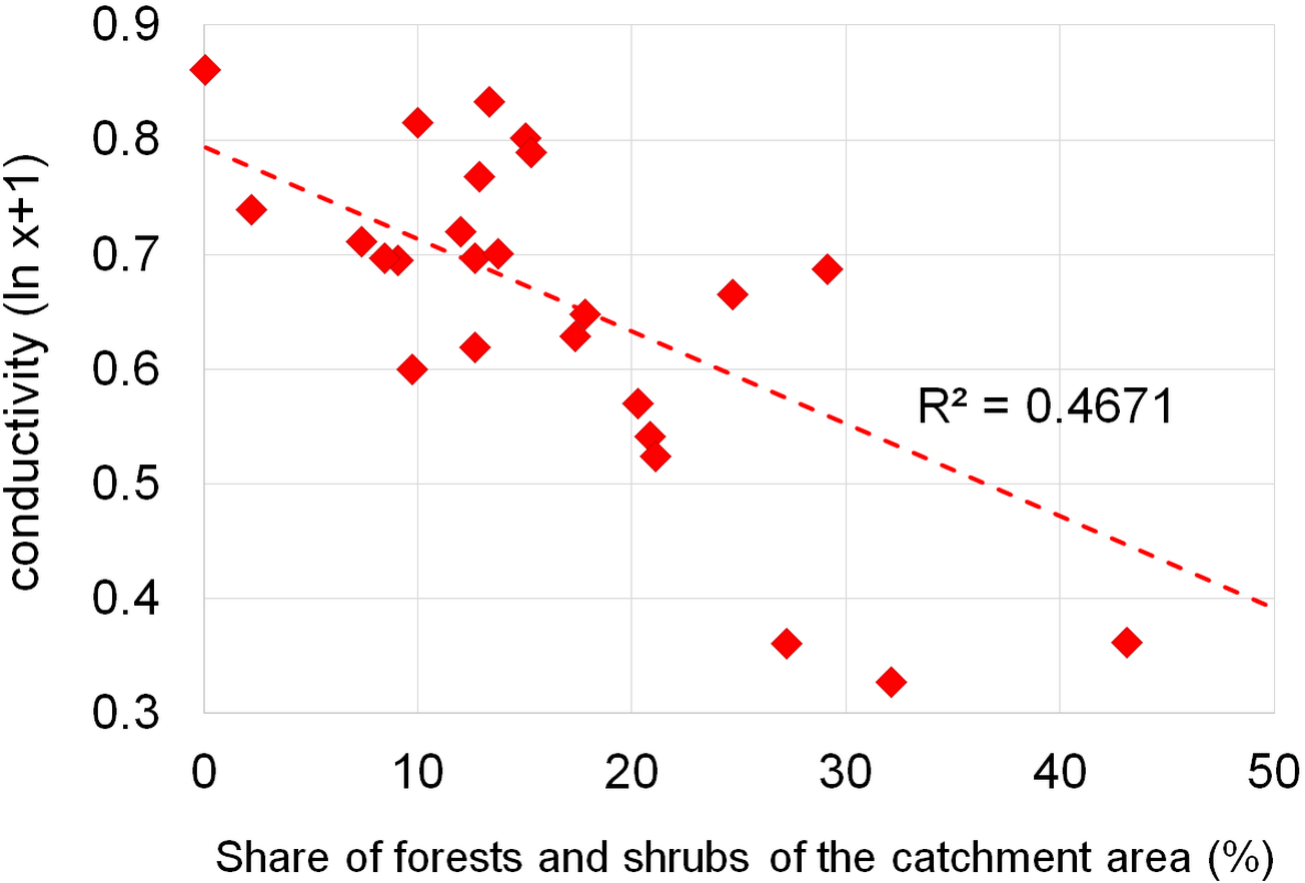
Dependencies between the forest and shrubs and electrolytic conductivity of water.

**Figure 9:**
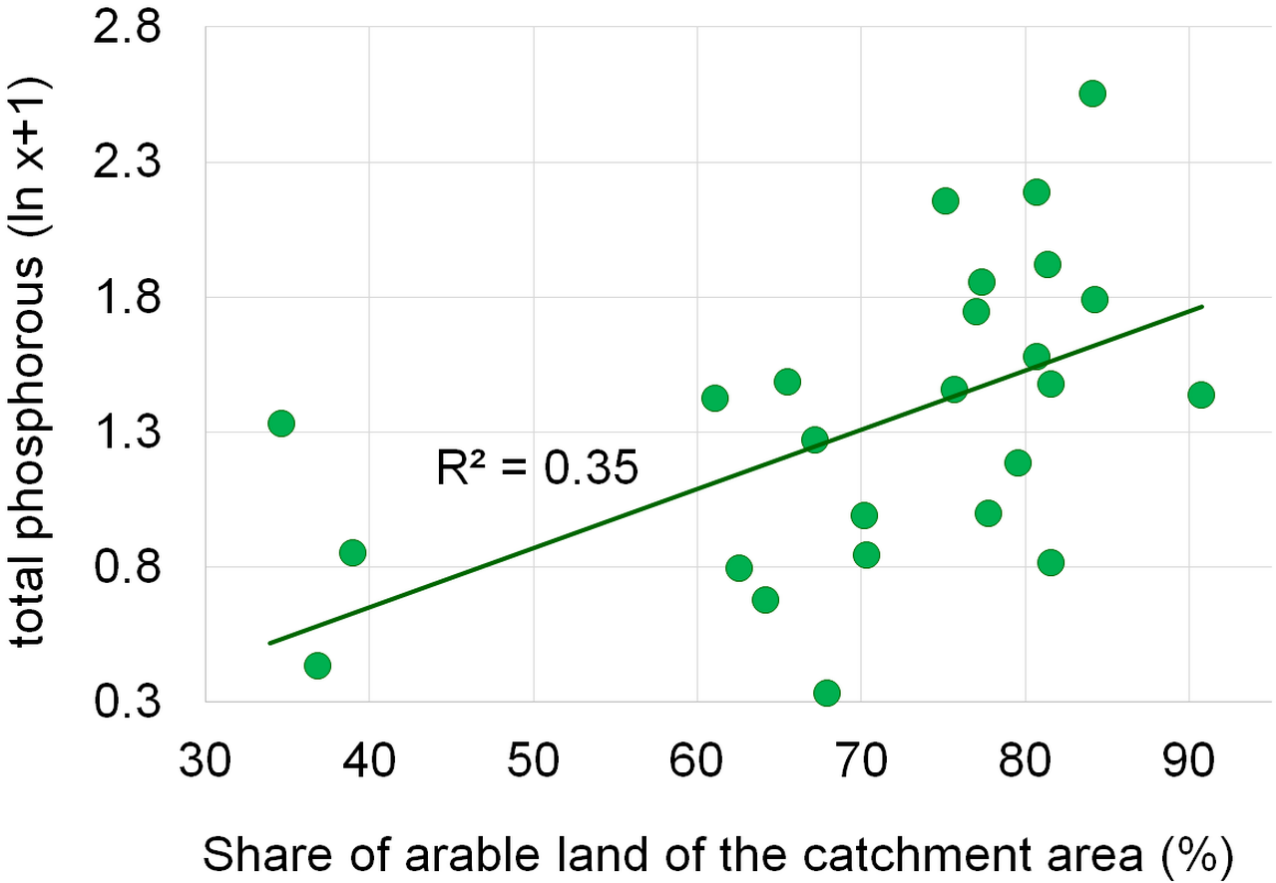
Dependencies between the arable land and concentration of total phosphorus.

**Figure 10:**
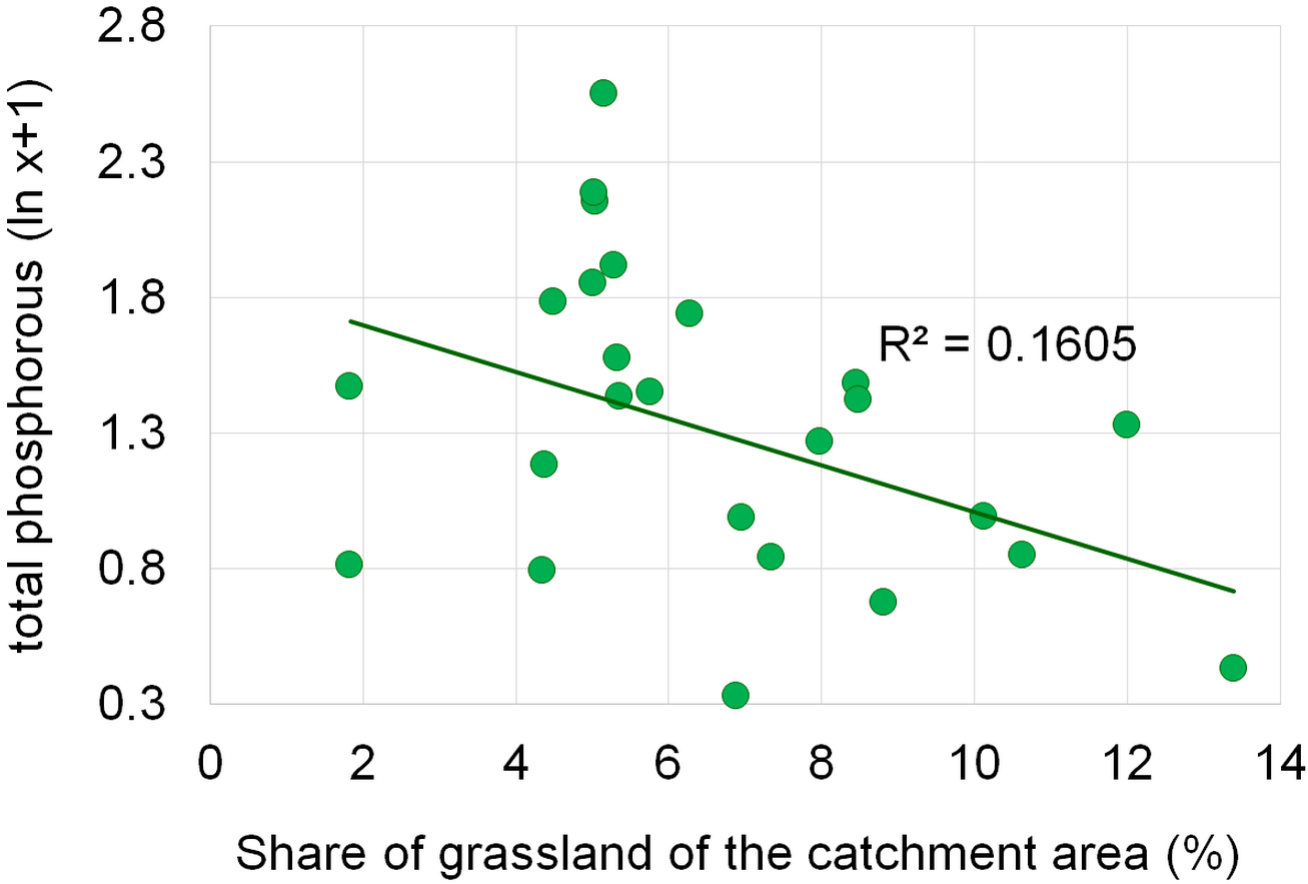
Dependencies between the grassland and concentration of total phosphorus.

**Figure 11:**
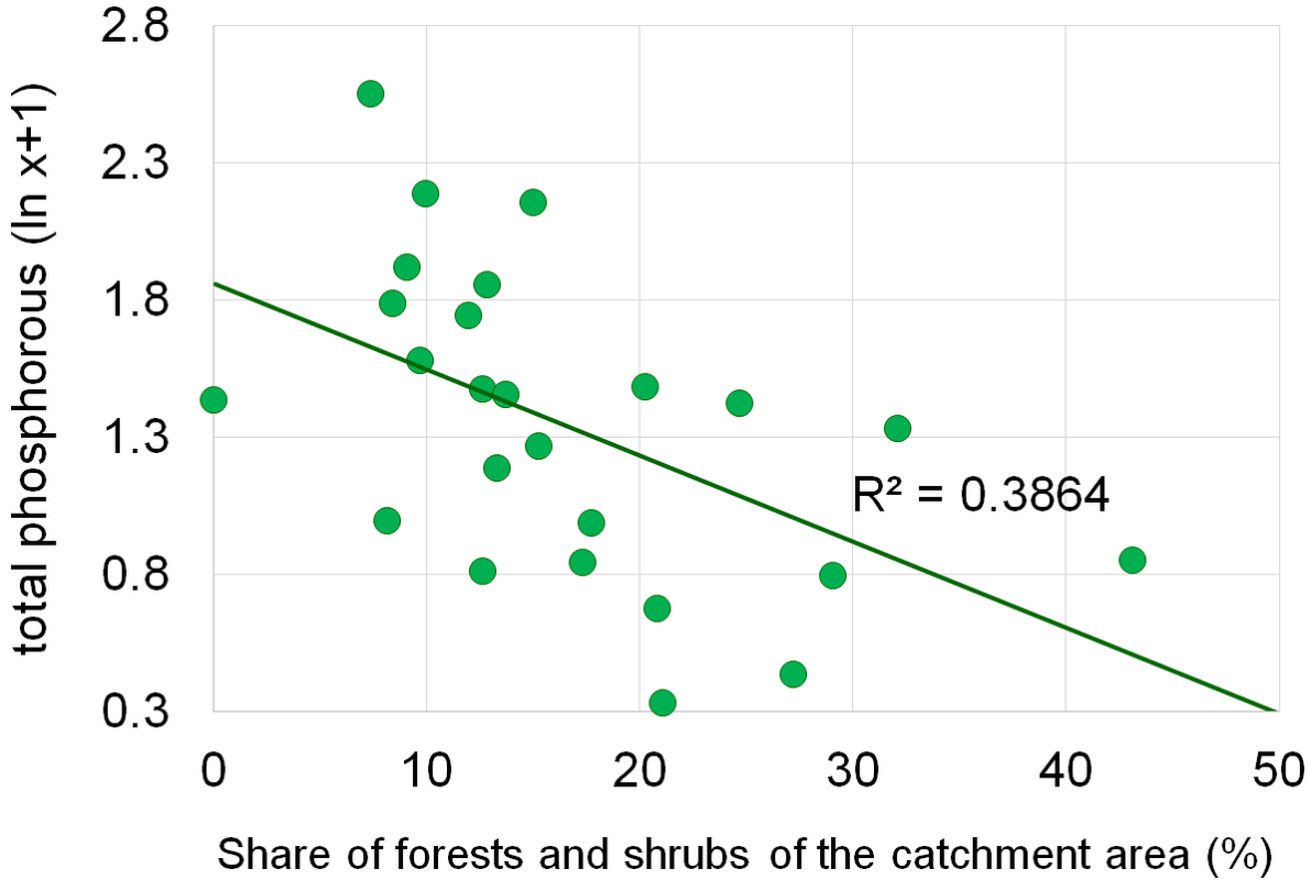
Dependencies between the forest and shrubs and concentration of total phosphorus.

**Figure 12:**
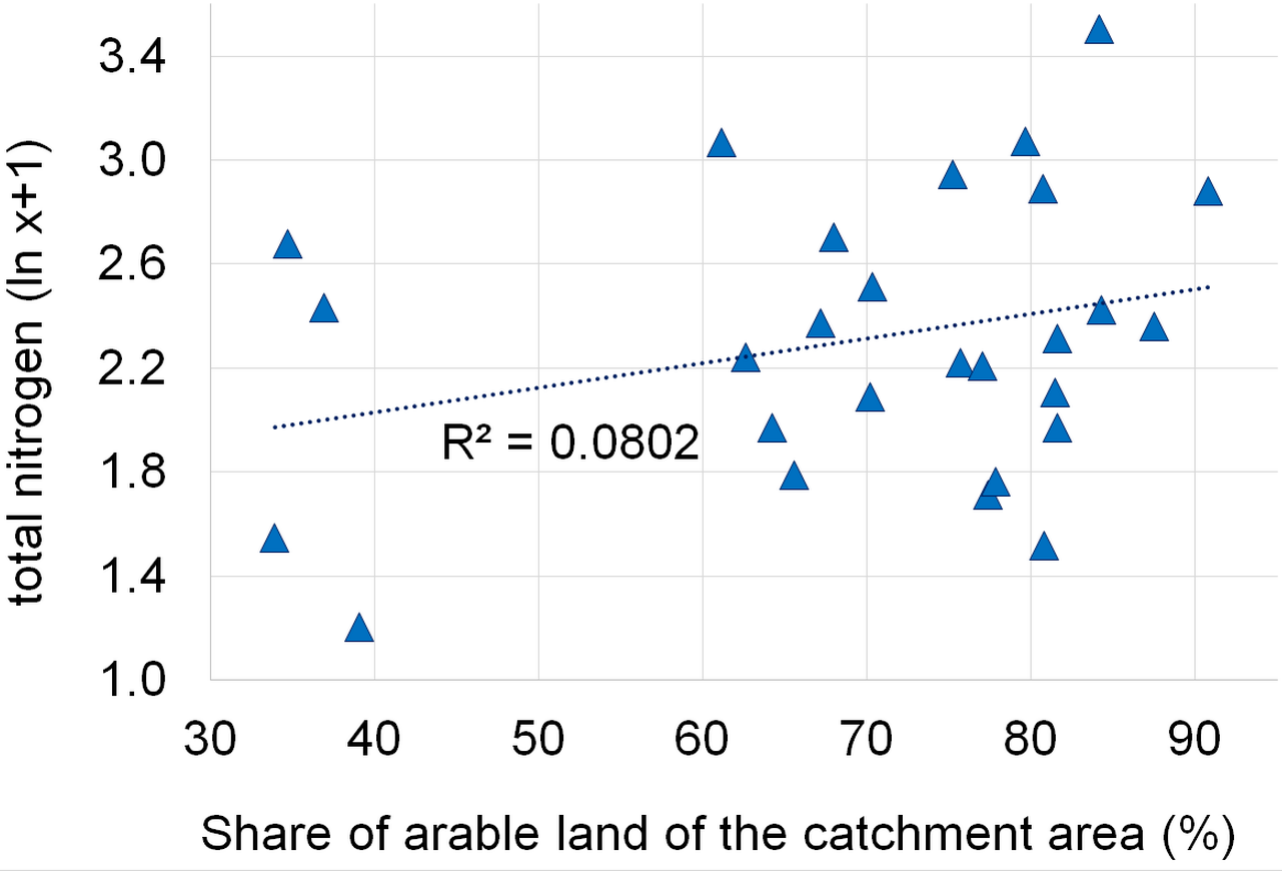
Dependencies between the arable land and concentration of total nitrogen.

**Figure 13:**
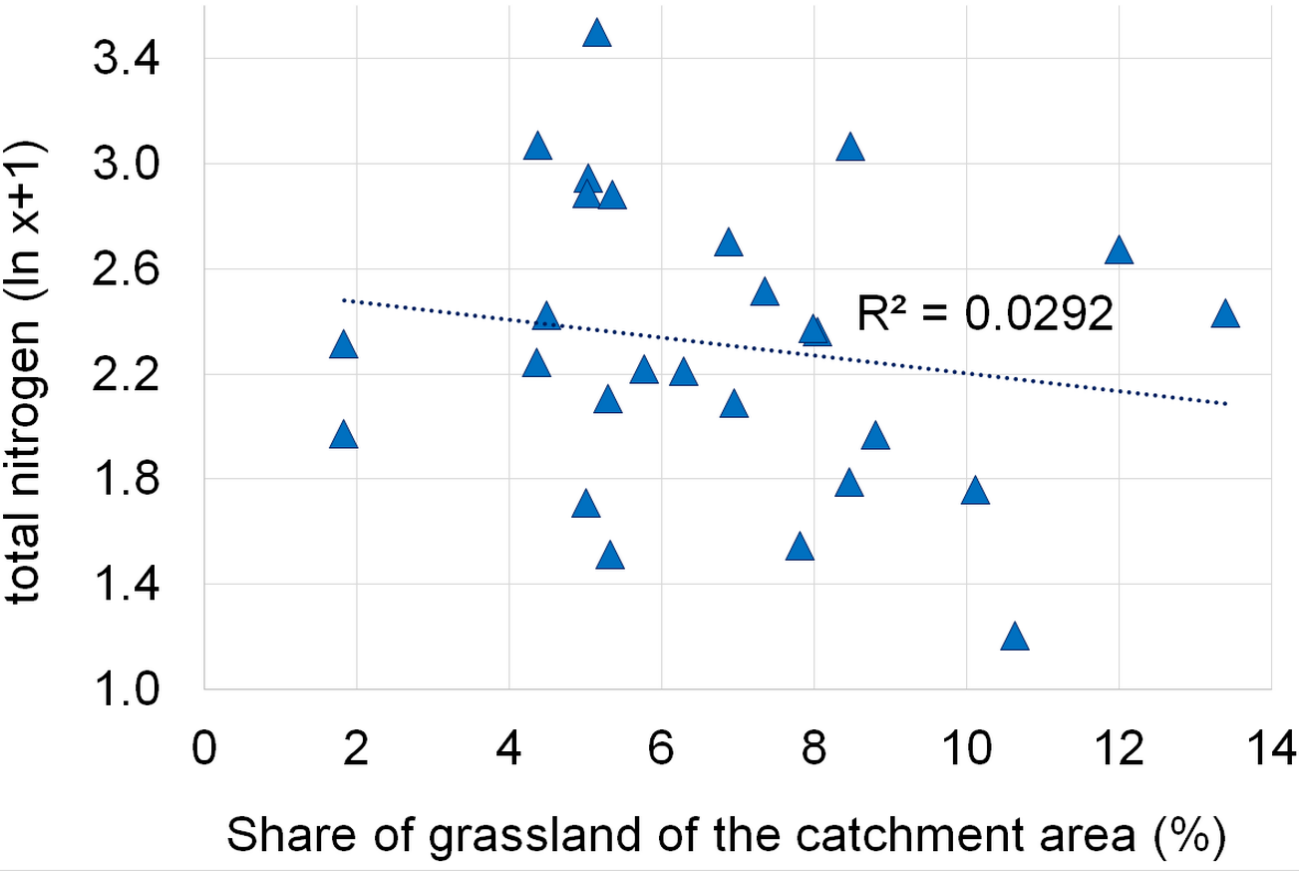
Dependencies between the grassland and concentration of total nitrogen.

**Figure 14:**
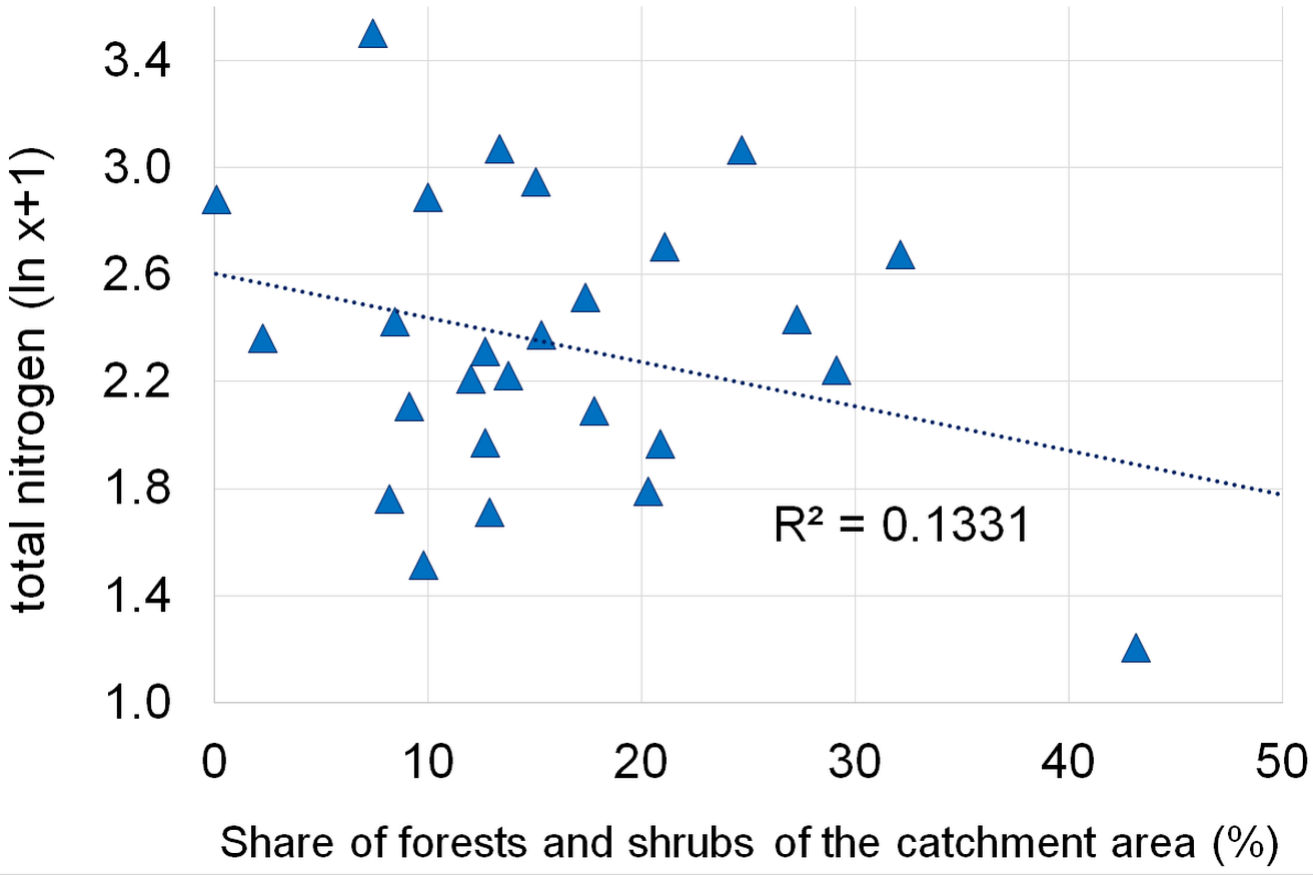
Dependencies between the forest and shrubs and concentration of total nitrogen.

Dependencies between the structure of use and the tested water quality parameters are presented in figures no 6–14. Results of the analyses indicate a strong effect of arable land on conductivity of flowing waters in the Orla catchment (Table 5). In most investigated river segments arable land accounted for 60–90% cover in the subcatchment. Their increased share correlated (*r* = 0.660) with conductivity expressed as ln. In turn, the presence of grassland (*r* = 0.498) as well as forests and shelterbelts (*r* = 0.467) correlated with lower conductivity values (Figs. 6–8). Similar dependencies as in the case of conductivity ln were observed for ln of total phosphorus, while the highest correlation coefficient was recorded for forests and shelterbelts (*r* = 0.386) (Figs. 9–11). Among the analyzed macrophyte indexes only the share of macroalgae was significantly correlated with environmental variables, i.e., nitrate nitrogen (*r* = 0.486), the share of arable land (*r* = 0.469), the share of forests (*r* =  − 0.425) and with conductivity (*r* = 0.389) (Figs. 12–14). A lack of other correlations resulted probably from the small gradient of water trophic parameters, as the entire Orla catchment is intensively agriculturally utilized and the waters are eutrophicated.

**Table 5:** Spearman’s correlation coefficients between analyzed parameters (marked correlation coefficients significant at *p* < 0.05; ns, non-statistically significant).

Environmental variables	Urbanized areas	Arable land	Grassland	Forests	Wetlands
conductivity	ns	0.626	−0.556	−0.642	−0.596
reactive phosphorus	ns	0.440	−0.477	−0.471	ns
total phosphorus	ns	0.546	−0.398	−0.607	ns
nitrate nitrogen	0.596	ns	ns	ns	ns

**Figure 15:**
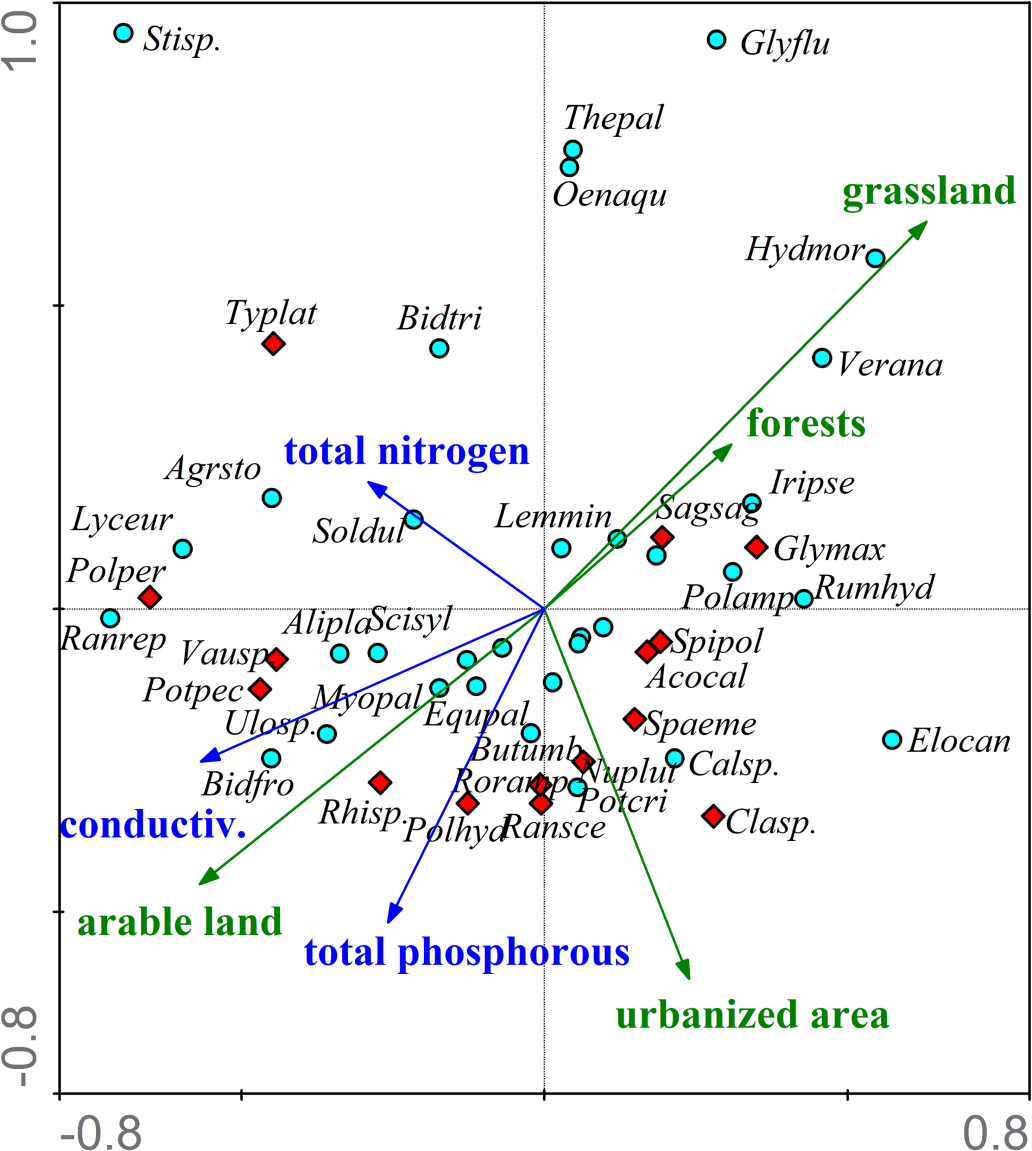
CCA ordination diagram of macrophyte species and environmental variables. The land use forms are marked with blue arrows, and the most important physicochemical parameters of the water are marked with turquoise arrows. Red dots represent macrophyte taxa, which are indicators of hypertrophy or strong eutrophy in at least two analyzed macrophyte indices (MIR, IBMR, RMNI, MTR, TIM, RI): Acocal—*Acorus calamus*, Clasp.—*Cladophora sp.*, Glymax—*Glyceria maxima*, Nuplut —*Nuphar lutea*, Polhyd—*Polygonum hydropiper*, Polper—*Polygonum persicaria*, Potpec—*Potamogeton pectinatus*, Ransce—*Ranunculus sceleratus*, Rhisp.—*Rhizoclonium sp.*, Roramp—*Rorippa amphibia*, Sagsag—*Sagittaria sagittifolia*, Spaeme—*Sparganium emersum*, Spipol—*Spirodela polrhiza*, Typlat —*Typha latifolia*, Vausp.—*Vaucheria sp.* Green dots represent taxa other macrophytes: Agrsto—*Agrostis stolonifera*, Alipla—*Alisma plantago-aquatica*, Bidfro —*Bidens frondosa*, Bidtri—*Bidens tripartita*, Butumb—*Butomus umbellatus*, Equpal—*Equisetum palustre*, Calsp.—*Callitriche sp.*, Elocan—*Elodea canadensis*, Glyflu—*Glyceria fluitans*, Hydmor—*Hydrocharis morsus-ranae*, Iripse—*Iris pseudacorus*, Lemmin—*Lemna minor*, Lyceur—*Lycopus europaeus*, Myopal —*Myosotis palustris*, Oenaqu—*Oenanthe aquatica*, Polamp—*Polygonum amphibium*, Potcri—*Potamogeton crispus*, Ranrep—*Ranunculus reprens*, Rumhyd—*Rumex hydrolapathum*, Scisyl—*Scirpus sylvaticus*, Soldul—*Solanum dulcamara*, Stisp.—*Stigeolonium sp.*, Thepal —*Thelypteris palustris*, Ulosp.—*Ulothrix sp.*, Vausp.—*Vaucheria sp.*, Verana—*Veronica anagallis-aquatica*.

The relationship between macrophyte taxa and environmental variables was demonstrated by CCA analysis and presented graphically (Fig. 15). The firrst axis consists of 29.4% (λ_1_ = 0.304) and the second axis for 20.9% (λ_2_ = 0.215) of the total variance in the relationships between the macrophytes and environmental variables. The first axis is the land use gradient related to the main physico-chemical parameters of the water. With the increase in the share of arable land in the catchment area, the conductivity and concentration of total phosphorus in the water also increases. On the other hand, the increase in the share of meadows and pastures as well as forests in the catchment area improves water quality. The second axis is related to urbanized areas (mainly buildings) and concentrations of total phosphorus in water. The Monte Carlo permutation test (Table 6) showed that only one variable presented significant marginal variance (λ_1_ > 0.20; *p* < 0.05) and conditional variance (λ_*A*_ > 0.05; *p* < 0.05). Macrophyte taxa that are indicators of eutrophication (red dots in Fig. 15) prefer river sections characterized by the highest conductivity and the highest concentrations of total phosphorus in the water. At the same time, these are the sections for which arable land and urbanized areas have the largest share in the structure of the catchment area use. This group includes three macroalgal taxa (*Cladophora sp.*, *Rhizoclonium sp.*, *Vaucheria sp.*) and several species of vascular plants (eg. *Potamogeton pectinatus*, *Ranunculus sceleratus*).

**Table 6:** Results of the Monte Carlo permutation test of the relationship between species composition and environmental variables.

Variables	Marginal effects	Conditional effects		
	λ_1_	λ_*A*_	P	F
grassland	0.23	0.23	0.020	1.79
arable land	0.19	0.17	0.092	1.36
conductivity	0.18	0.14	0.276	1.18
urbanized areas	0.16	0.12	0.480	0.98
total phosphorous	0.15	0.17	0.114	1.36
forests and shrubs	0.14	0.11	0.592	0.89
total nitrogen	0.12	0.09	0.742	0.78

**Notes.**

λ_1_ is the proportion of variance explained by each single environmental variable, and λ_*A*_ is the proportion of conditional variance explained by the variable in forward selection.

## Discussion

Appropriate catchment management promotes protection of water resources and facilitates utilization of river valleys for ecological and recreation purposes (Hua, 2017). Changes towards uncontrolled urbanization may considerably increase the intensity of threats such as soil erosion and as a consequence lead to the deterioration of river water quality and habitat fragmentation (Hua, 2015). A high share of arable land and a low share of forests had a direct effect on water quality in the Orla River, as indicated not only by chemical, but also biological parameters (macrophytes). Excessive nitrate concentrations in aquatic ecosystems may result e.g., in a reduction of biodiversity as a consequence of depletion of plant species from poor habitats (Jasiewicz & Baran, 2006). Long-term studies conducted over many years indicate that excessive nitrate concentrations are cyclical. This may result in their potential negative effect on the diversity of macrophytes and the presence of specific ecological groups.

Land use in the catchment has a marked effect on quality of surface waters. A study by Haidary et al. (2013) showed a statistically significant dependence between an increased area of agricultural land in the catchment and an increase in water conductivity of wetlands in Japan. In the case of forests the correlation is negative, while for meadows it was close to zero. A similar trend was also observed in this study. An increase in the share of arable land resulted in an increase of water conductivity. A considerable effect of agriculturally utilised areas on quality of river waters was observed in many studies.

Changes in land use affect physical and chemical parameters of surface waters (Rawicki et al., 2015; Afed Ullah, Jiang & Wang, 2018; Szatten & Habel, 2020). Modifications of the land use structure in the Rzyki river catchment influenced concentrations of nitrate and ammonia nitrogen. A decrease in the area of arable land by 69% resulted in a 4-fold reduction of nutrient concentrations (Kowalik et al., 2015). Li et al. (2019) found negative correlation between presence of forests and grasses in watershed and values of trophic parameters, total phosphorus and total nitrogen.

Walsh & Wepener (2009) when analyzing water quality indexes in Southern Africa showed that in areas with intensive farming river waters fall into water quality classes ranging from poor to moderate. At the same time they stated that the species composition of diatoms in urbanized areas corresponded to waters of moderate purity class. Skinner et al. (1997) and Walsh & Wepener (2009) pointed to the potential increased runoff of biotic components from modern agriculture and a deterioration of quality in waters adjacent to agricultural areas, also due to morphological transformations of river channels. Both the Orla and its tributaries are considerably transformed by anthropogenic pressure. The catchment area is one of the most intensively utilized agricultural areas in Poland (Majchrzak, 2008; Central Statistical Office, 2020).

In turn, Clerici & Vogt (2013) and Clerici, Paracchini & Maes (2014) analyzed available data for watercourse bank zones in Europe taking into consideration only natural and semi-natural segments and the 500-m wide littoral zone. Those authors stated that land use also affects considerably the service aspects of watercourse littoral zones. Clerici, Paracchini & Maes (2014) showed that the presence of wetlands, forested areas as well as vegetation other than forest vegetation have an impact on water self-purification, intensity of erosion processes and reduction of biotic components influx. Moreover, the presence of forests contributes to the regulation of local climatic conditions and facilitates flood control. An advantageous effect of shelterbelts and self-purification of rivers draining forested areas was also reported in other studies, e.g., Krzemińska, Adynkiewicz-Piragas & Kazimierska (2006) and Staniszewski et al. (2017). Similar trends were also observed in this study. The southern part of the Orla catchment, characterized by a greater share of forests and surface waters, showed more advantageous environmental parameters (both chemical and biological).

In turn, Zamani, Sadoddin & Garizi (2013) analyzed the effect of changes in land use structure in the Ziarat river catchment (Iran) on quality of river waters and reported such an impact on the quality of flowing waters. In the case of electrolytic conductivity of waters an upward trend was shown, correlated with an increase in agriculturally utilized areas (from 18% in 1967 to 24.8% in 2012) and a decrease in forested areas (from 79.9% in 1967 to 69.8% in 2012).

Rosso & Fernandez Cirelli (2013) investigated macrophytes in watercourses located in the Rio de la Plata Grasslands (Argentina), characterized by the predominance of arable land in the catchment or intensive animal production. They stated the impact of arable land present in the region on a reduction of pH and an increase in nitrate nitrogen concentration in waters. Waters with lower concentrations of total phosphorus were characterized by a greater macrophyte cover. Higher concentrations of total phosphorus were recorded in catchments with the dominance of arable land. Conductivity was also greater in the areas with intensive animal production. The Orla catchment is exposed to the pressure of animal production. Apart from family farms applying intensive farming systems in the catchment there are also several very large commercial farms with large-scale animal production. Ritter (2001) and Foy & O’Connor (2002) when investigating point pollution sources in agricultural regions stated that they influence the quality of groundwater and waters in the watercourses located in their immediate vicinity.

Gebler & Szoszkiewicz (2011) showed a dependence between MIR and eutrophication in catchments located in agricultural regions. The complexity of agricultural operations prevents accurate and precise identification of the number of sources and volumes of pollutant emissions (Moss, 2008), thus formulating of precise conclusions is difficult. They can be formulate predominantly for small subcatchments with similar land use structure and lack of major point sources of pollution (Staniszewski et al., 2019).

It was observed in other studies (Gocić et al., 2020), that intensive land use along watercourses intensified erosion processes and can intensified surface and subsurface runoff from permeable soils that dominate in this region. It can accelerate river degradation processes in the catchment. The biggest challenge in studied and similar areas is changing the management of the coastal zone and its tributaries. The introduction of ecotone zones is very important, and in the most sensitive places also the solutions proposed by Izydorczyk et al. (2015). The introduction of trees and shrubs along the banks will initiate the diversity of the vegetation structure in the riverbed, but will also have a positive effect on the hydromorphology of the watercourse and biodiversity. Regulatory work, especially regarding profiling the banks and bottom of the riverbed, should be carefully planned to avoid additional problems related to unstability of sediments (Prus, Popek & Pawlaczyk, 2018).

## Conclusions

1. Surface waters in the Orla catchment are strongly eutrophicated. This is caused both by point pollution (2.394 million m^3^/year sewage, approx. 24.1% ALD) and surface pollution from agricultural sources (the 64% share of arable land).
2. The highest exceeding of water quality thresholds were observed in case of orthophosphates (10.7 times in the Orla River and 10.4 times in its tributaries) and ammonia nitrogen (6 times and 12 times respectively).
3. In the investigated catchment water conductivity was observed to increase with an increase in the arable land area. An opposite trend was found in the case of grassland or forests and shelterbelts.
4. An increase was also recorded in the concentration of total phosphorus with an increase in the area covered by arable land, although this correlation was less significant than in the case of conductivity.
5. The worst ecological status (poor) among the investigated sites was reported for two right bank tributaries of the Orla, i.e., the Dąbrocznia and Masłówka were share of arable lands was the highest. Calculated macrophyte indices confirmed strong eutrophication of studied river sites and the problem of failure to achieve good ecological status was observed.
6. In the analyzed watercourses common macrophyte species predominated, including many taxa indicating strong eutrophication of waters, e.g., *Ceratophyllum demersum*, *C. submersum*, *Cladophora sp.*, *Lemna gibba*, *L. minor*, *Potamogeton pectinatus*, *Ranunculus sceleratus*, *Rhizoclonium sp.*, *Spirodela polyrhiza*, *Stigeoclonium sp.*, *Typha latifolia* and *Vaucheria sp.* Plant cover of mentioned species was the highest in areas with greater share of arable lands.

##  Supplemental Information

10.7717/peerj.10564/supp-1 Supplemental Information 1 Macrophytes data A list of identified macrophyte species and the calculated biodiversity indicators for individual points.

10.7717/peerj.10564/supp-2 Supplemental Information 2 Macrophytes indexes, physical and chemicals parameters of water and landuse of Orla catchment

## Additional Information and Declarations

### Data Availability

The following information was supplied regarding data availability: Raw data, including data related to the biodiversity of macrophytes, their coverage, but also for land use in catchments and partial catchments, physical and chemical state of water and loads of selected parameters from sewage treatment plants operating in the catchment, are available in the Supplementary Files.
